# *In vitro* evaluation of the interaction between orbital fibroblasts and PBMCs from Graves’ orbitopathy patients in triggering periorbital remodeling

**DOI:** 10.3389/fimmu.2026.1728194

**Published:** 2026-09-09

**Authors:** Aleksandra Starosz, Willem A. Dik, Dion Paridaens, J. Conny P. A. van Holten Neelen, Stéphanie Goes, Przemysław Pawłowski, Kamil Grubczak

**Affiliations:** 1 Department of Regenerative Medicine and Immune Regulation, Medical University of Bialystok, Bialystok, Poland; 2 Laboratory Medical Immunology, Department of Immunology, Erasmus University Medical Center, Rotterdam, Netherlands; 3 Laboratory Medical Immunology, Reinier Haga Medisch Diagnostisch Centrum (RHMDC), Delft, Netherlands; 4 Department of Oculoplastic, Lacrimal and Orbital Surgery, Rotterdam Eye Hospital, Rotterdam, Netherlands; 5 Department of Ophthalmology, Erasmus Medical Center (MC) University Medical Center Rotterdam, Rotterdam, Netherlands; 6 Department of Paediatric Ophthalmology and Strabismus, Medical University of Bialystok, Bialystok, Poland; 7 Department of Medical Pathology, Cathedral of Biostructure, Medical University of Bialystok, Bialystok, Poland; 8 Department of Allergology and Internal Medicine, Medical University of Bialystok, Bialystok, Poland

**Keywords:** Graves’ orbitopathy, immune cells, immune modulation, orbital fibroblasts, remodeling

## Abstract

**Background:**

Graves’ orbitopathy (GO) is a complication of Graves’ disease characterized by periorbital tissue inflammation, adipose tissue expansion, and fibrosis. The infiltration of immune cells into the periorbital tissue is thought to be a crucial factor in triggering the complication. Although orbital fibroblasts are considered to represent key cellular elements in the pathogenesis of GO, their interaction with immune cells is hardly explored. Here, we aimed to study *in vitro* the effects of autologous peripheral blood mononuclear cells (PBMCs) on orbital fibroblasts derived from GO patients with active/inactive disease with or without steroids and vitamin D3.

**Methods:**

Orbital fibroblasts and PBMCs collected from active and inactive GO patients were co-cultured for 48 h in the presence of vitamin D3 and/or steroids (methylprednisolone, MP). Flow cytometric, quantitative PCR (qPCR), and immunoenzymatic assessments enabled evaluation of the changes during cell-to-cell interactions.

**Results:**

We found that orbital fibroblasts from inactive GO exhibited higher expression of the genes associated with tissue remodeling. Co-culture of fibroblasts with PBMCs enhanced the proliferation rate of fibroblasts, especially the fibroblasts from active GO. Moreover, the cytokine secretion patterns and early changes in the deposition of extracellular matrix compounds by orbital fibroblasts were also found to differ between disease stages. In addition, we demonstrate that exposure to MP reduces fibroblast proliferation, particularly those derived from the inactive disease stage, and that the combination of MP with vitamin D3 suppresses IL-6 and CCL-20 secretion.

**Conclusion:**

We propose that the pathogenesis of GO involves mutual interactions between orbital fibroblasts and immune cells, which may be disease stage-specific and can be modulated by MP and vitamin D3.

## Introduction

Graves’ disease (GD) is an organ-specific autoimmune disorder characterized by abnormal thyroid function due to the presence of stimulatory thyroid-stimulating hormone (TSH) receptor autoantibodies (1). GD is the most common cause of hyperthyroidism (with an annual adjusted incidence rate of 16 cases per 100,000 women and 3 cases per 100,000 men) (2, 3). Approximately 50% of patients with Graves’ hyperthyroidism develop Graves’ orbitopathy (GO), with symptoms such as eye dryness, tearing, diplopia, and retrobulbar pressure. GO, which is also referred to as thyroid eye disease (TED), is most commonly associated with GD, but may also occur in patients with other autoimmune thyroid diseases, including Hashimoto’s thyroiditis (4). Typical signs include eyelid retraction, periorbital inflammation, and proptosis. Severe GO (~3%–5%) involves marked, intense pain, inflammation, tissue remodeling, and sight-threatening complications such as compressive optic neuropathy (5, 6). Based on the current knowledge, GO onset is influenced by various factors, including immunological, genetic, and environmental causes (7). The presence of TSH receptors on orbital fibroblasts (OFs) indicates their key role in the pathogenesis of GO and underpins potential targeted therapies (8, 9).

The orbital manifestation of GO primarily results from tissue enlargement due to fibroblast proliferation and adipogenesis. Disease progression follows two clinical stages—active and inactive—categorized using the Clinical Activity Score (CAS) (4). These phases reflect distinct fibroblast activity: the active phase is marked by inflammation and fibroblast activation, whereas the inactive phase is characterized by persistent tissue remodeling and accumulation of extracellular matrix (ECM) components such as hyaluronan and collagen (10). Over time, GO transitions from an active to an inactive phase as inflammation and cellular infiltration decrease (5).

OFs are central to the disease pathogenesis of GO through dynamic and mutual interactions with infiltrating immune cells. Fibroblasts not only act as targets of immune activation but also as active participants in shaping the inflammatory microenvironment (10). OFs respond to cytokines from infiltrating T cells and macrophages by releasing pro-inflammatory mediators including interleukin 6 (IL-6) and IL-8, amplifying inflammation, and secreting ECM components such as hyaluronan and collagen, thereby promoting tissue expansion and fibrosis (10). Previous studies have demonstrated that autologous activated T lymphocytes stimulate OF proliferation through contact-dependent mechanisms involving MHC class II and CD40–CD40L signaling, highlighting the importance of direct immune cell–fibroblast interactions in the pathogenesis of GO (11). Wang et al. highlighted the role of cell-mediated immunity in GO, particularly that of T helper 1 (Th1) and Th17 cells in modulating fibroblast responses. Moreover, fibroblasts themselves can express HLA-DR and co-stimulatory molecules, facilitating further T-cell activation (12). Huang et al. further emphasized that the bidirectional crosstalk between OFs and T cells, in which immune-derived cytokines promote fibroblast proliferation and their differentiation into adipocytes or myofibroblasts, is crucial to the characteristic tissue remodeling in GO (13).

CD4^+^ Th lymphocytes are predominant immune cells that infiltrate GO orbital tissue. However, other immune cells, including CD8^+^ T lymphocytes, B lymphocytes, mast cells, and macrophages, also contribute to the infiltrate (14). In active GO, Th1 lymphocytes, which secrete, among others, high amounts of interferon gamma (IFN-γ) and tumor necrosis factor alpha (TNF-α), strongly drive the inflammatory process. As the disease progresses, Th1 lymphocytes are gradually replaced by Th2 lymphocytes, which promote autoantibody production and tissue remodeling (15, 16). Recent studies have further enhanced our understanding of immune cell involvement in GO and demonstrated that the onset of orbitopathy involves infiltration by a variety of immune cells, including regulatory T lymphocytes (Tregs), effector T lymphocytes (particularly Th17 cells), plasmablasts, and dendritic cells (13). Moreover, Th17.1 cells that produce IFN-γ and IL-17A are markedly elevated in moderate-to-severe and corticosteroid-resistant GO, implying that their cooperative cytokine activity is crucial to the disease pathogenesis (17). Cytotoxic CD4^+^ lymphocytes can also contribute to orbital inflammation and orbital tissue remodeling in GO (18). Furthermore, Görtz and colleagues demonstrated that TNF-α-secreting macrophages stimulate OFs to release chemokines, thereby perpetuating a pro-inflammatory state and promoting further immune cell infiltration (19). However, the cytokines produced by resident cells, including OFs and adipocytes, together with infiltrating immune cells, orchestrate immune responses and tissue remodeling. Orbital recruitment of immune cells triggers cytokine-dependent activation of OFs, leading to glycosaminoglycan accumulation and tissue expansion, and further promotes tissue remodeling (20, 21). Chemokines also influence immune cell infiltration. They regulate immune cell recruitment, inflammation, and interactions with resident cells, such as OFs. Th1-associated chemokines such as CXCL9, CXCL10, and CXCL11 can act via CXCR3 to recruit immune cells, in particular T lymphocytes, into thyroid and orbital tissues. Moreover, thyroid follicular cells and other resident cells produce CXCL9, CXCL10, and CXCL11 in response to IFN-γ, inducing the migration of CXCR3^+^ lymphocytes. In early-stage orbitopathy, Th1-mediated cytokines drive OF proliferation and glycosaminoglycan synthesis, while IFN-γ induces chemokine-mediated lymphocyte recruitment. Hypoxia and macrophages can further stimulate OFs to produce inflammatory mediators, including CCL2, CCL5, CCL20, and TNF-α (22).

Vitamin D has immunomodulatory properties that regulate both the innate and adaptive immune responses through its receptor (vitamin D receptor, VDR), which is expressed in immune cells and fibroblasts. Recently, it has been shown to suppress the pro-inflammatory Th1 and Th17 pathways while promoting regulatory immune mechanisms. Its deficiency has been associated with increased disease activity in autoimmune thyroid disorders, including GD. Moreover, vitamin D may directly influence OF function by reducing cytokine production, thereby modulating local inflammatory responses within the orbit (23–25).

The current first-line treatment for GO involves high doses of steroids to mitigate inflammatory infiltration. However, chronic systemic intake of large steroid doses can be associated with a significant risk of serious side effects. Moreover, while steroids can alleviate symptoms, they do not address the underlying cause of the disease, and prolonged use may lead to adverse complications (26, 27). In this study, we aimed to evaluate the effect of the corticosteroid methylprednisolone (MP) in an *in vitro* co-culture model established with OFs and matched autologous immune cells from patients with GO. Moreover, we investigated whether the observed biological responses to MP are related to the disease stage. In addition, we aimed to determine whether lowering the MP concentration, along with vitamin D3 supplementation, could help reduce inflammation and fibrosis.

## Materials and methods

### General characterization of the studied groups

Periorbital tissue and peripheral blood from patients with GO (*n* = 5 per group) were collected from the same donors during orbital decompression surgery conducted at the Rotterdam Eye Hospital (Rotterdam, the Netherlands). Materials were further processed at the Laboratory of Medical Immunology, Department of Immunology, Erasmus Medical Center (Rotterdam, the Netherlands). The obtained tissue was washed and the OFs isolated and cryopreserved in liquid nitrogen, as previously described (28). Peripheral blood mononuclear cells (PBMCs) were isolated from the whole blood sample using Ficoll density gradient centrifugation and then stored in liquid nitrogen. The OFs and PBMCs used for this study were shipped on dry ice to the Department of Regenerative Medicine and Immune Regulation, Medical University of Bialystok, Poland, where they were stored in liquid nitrogen until the start of the study.

Active GO was defined as a total CAS of ≥3 in one or both eyes. The clinical data of the patients are given in **Supplementary Tables 1A, B**. The study protocol was approved by the Medical Ethical Committee at Erasmus MC (MEC-2007-124) and the Ethical Committee at the Medical University of Bialystok (APK.002.84.2021).

### Orbital fibroblast culture

Vials with OFs were thawed (at 37°C), and the cell suspension was transferred into a culture medium (Dulbecco’s minimum essential medium, DMEM), supplemented with 10% fetal bovine serum (FBS) and gentamicin (PAN Biotech, Aidenbach, Germany), and cultured in a humidified 5% CO_2_ incubator at 37°C. The OFs used for the experiments were from the third passage.

### Exposure of orbital fibroblast–PBMC co-cultures to methylprednisolone and vitamin D3

The OFs were seeded at 4–5 × 10^4^ cells/well onto 24-well plates. Subsequently, to the part of the wells with OFs, strictly matched autologous PBMCs (1 × 10^5^ PBMCs/well) were added. In addition, for selected wells, MP at concentrations of 0.2 and 1.0 μM (methylprednisolone sodium succinate; Solu-Medrol, Pfizer, New York, NY, USA) was added, either alone or in the presence of an active form of vitamin D (calcitriol, at a concentration of 100 nM) (1,25-dihydroxycholecalciferol; Cayman Chemicals, Ann Arbor, MI, USA). All culture conditions were incubated for 48 h.

Following incubation, supernatants containing non-adherent cells (PBMCs) were aspirated and centrifuged to obtain cells and supernatants for the next part of the study. OFs were washed twice with phosphate-buffered saline (PBS) and trypsinized (0.25% trypsin; Sigma-Aldrich, St. Louis, MO, USA). Detached cells were suspended in DMEM containing 10% FBS to inactivate trypsin, washed in PBS, and used for the flow cytometric analysis. In addition, a portion of the OFs was subjected to lysing with RLT buffer (Qiagen, Hilden, Germany) supplemented with β-mercaptoethanol (Sigma-Aldrich, St. Louis, MO, USA) for further RNA isolation (stored at −80°C).

### Flow cytometric assessment of orbital fibroblast proliferation rate

The proliferative activity of OFs was determined using CFSE [5(6)-carboxyfluorescein diacetate *N*-succinimidyl ester; Sigma-Aldrich, St. Louis, MO, USA] staining along with live/dead staining with 7AAD (BD Pharmingen, San Jose, CA, USA). As a negative control, colcemid (25 ng/ml; Biowest, Nuaille, France) was added to the OFs. To exclude the possibility of PBMC contamination, we additionally stained the cells with anti-CD45 PE (clone HI30; BD Biosciences, Franklin Lakes, NJ, USA) and only analyzed CD45-negative cells. Measurement was conducted on a FACS Calibur flow cytometer (BD Biosciences, Franklin Lakes, NJ, USA), and subsequent analysis was performed using FlowJo® software (TreeStar Inc., Ashland, OR, USA).

### Flow cytometric immunophenotyping of immune cells

Matched PBMCs derived from patients with GO were stained with fluorochrome-conjugated anti-CD4 FITC (clone RPA-T4; BD Biosciences, Franklin Lakes, NJ, USA) for 25 min and then washed with PBS (Corning, Corning, NY, USA). For intracellular staining of IL-17A and Foxp3, the cells were permeabilized (Permeabilization Buffer 2; BD Biosciences, Franklin Lakes, NJ, USA) for 7 min, washed, and stained with anti-IL-17A PE (clone SCPL1362) and anti-Foxp3 Alexa Fluor 647 (clone 259D/C7) (both from BD Biosciences, Franklin Lakes, NJ, USA). Subsequently, the cells were washed (PBS), fixed with CellFix Buffer (BD Pharmingen, San Jose, CA, USA), and stored briefly at 4°C until measurement. Measurement was conducted on a FACS Calibur flow cytometer (BD Biosciences, Franklin Lakes, NJ, USA), and subsequent analysis was performed using FlowJo® software (TreeStar Inc., Ashland, OR, USA).

Lymphocytes were gated based on the forward scatter (FSC) and side scatter (SSC) profile, and further based on the presence of the CD4^+^ surface marker. Evaluation of the levels of IL-17A and Foxp3 in CD4^+^ lymphocytes included the frequencies of IL-17A- and Foxp3-expressing cells and the mean fluorescence intensities (MFIs) in these cells.

### Cytokine measurement in the culture supernatant

Supernatants from the co-cultures were collected from designated wells, and the limits of detection (LODs) of the cytokines [IL-1β: LOD = 3.9–250 pg/ml; IL-6: LOD = 9.4–600 pg/ml; IL-17: LOD = 15.6–1,000 pg/ml; TNF-α: LOD = 15.6–1,000 pg/ml; TGF-β: LOD = 31.2–2,000 pg/ml; and CCL-20: LOD = 15.6–1,000 pg/ml] were measured with ELISA (all DuoSet ELISA kits from R&D Systems, Minneapolis, MN, USA) according to the manufacturer’s protocol. As controls, PBMCs and OFs were cultured separately for 48 h under experimental conditions identical to those of the co-cultures, and the cytokine concentrations in the culture supernatants were determined. Standard curves based on a four-parameter logistic (4-PL) curve fit were used to calculate the cytokine concentrations.

### RT-PCR-based assessment of remodeling-related genes

After culture, messenger RNA (mRNA) was isolated using RNeasy Micro Kits (Qiagen, Hilden, Germany) according to the manufacturer’s protocol. The amount of mRNA was quantified on NanoDrop (Thermo Fisher Scientific, Waltham, MA, USA) and subsequently converted into complementary DNA (cDNA) using the High-Capacity cDNA Reverse-Transcription Kit (Applied Biosystems, Foster City, CA, USA) according to the manufacturer’s protocol with the following stages: (10 min, 25°C), (120 min, 37°C), and (5 min, 85°C).

The genes tested were assigned into the following groups of biological processes: 1) genes involved in ECM and its remodeling; 2) genes involved in the regulation of fibrosis; and 3) genes involved in response to hypoxia (**Supplementary Table 2**). The PCR stages were as follows: one cycle of uracil-*N*-glycosylase (UNG) incubation (50°C for 2 min), one cycle of polymerase activation (95°C for 10 min), 40 cycles of denaturation (95°C for 15 s), and annealing with elongation (60°C for 60 s). Assays were performed using the StepOnePlus Real-Time PCR System device (Applied Biosystems, Foster City, CA, USA) and analyzed with the manufacturer’s software (StepOnePlus v2.3). Data are presented as a relative expression log_2_(2^−Δ^*^C^*^t^).

### Biostatistical assessment

Statistical analyses were performed using GraphPad Prism 9.0 (GraphPad Software, San Diego, CA, USA). Baseline comparisons between independent groups (active *versus* inactive GO) were performed using the two-tailed Mann–Whitney *U* test. Data obtained from the co-culture experiments, comprising multiple experimental conditions evaluated within the donor-derived samples, were analyzed using a mixed-effects model followed by Fisher’s least significant difference (LSD) multiple comparisons test. The experimental unit was the individual donor. Data are presented as median with interquartile range. Statistical significance was accepted at *p* < 0.05, and differences are indicated with asterisks or exact *p*-values: *p < 0.05, **p < 0.01, ***p < 0.001, ****p < 0.0001.

## Results

### Basal characteristics of orbital fibroblasts from active and inactive GO patients

Firstly, we assessed “baseline” differences between the OFs and PBMCs from patients in the active or the inactive GO disease stage. The proliferative activity of the fibroblasts collected from the two different GO disease stages was comparable (**Supplementary Figure 1A**). Assessment of selected genes related to tissue fibrosis, ECM, and metalloproteinases (matrix metalloproteinases, MMPs) and their inhibitors (tissue inhibitors of metalloproteinases, TIMPs) revealed differences between the OFs obtained from active GO *versus* inactive GO. The OFs from inactive GO showed significantly higher expression of *aSMA* (alpha-smooth muscle actin; *p* = 0.0084) and *VTN* (vitronectin; *p* = 0.0454) than the OFs from active disease. On the contrary, the OFs from active disease exhibited higher expression levels of *MMP9* (matrix metalloproteinase-9; *p* = 0.0023) (**Supplementary Figure 1C**).

The levels of cytokines released did not differ significantly between the OFs obtained from active GO and inactive GO. However, the median values of IL-1β, IL-17, and TNF-α did show a trend to higher levels in the case of active GO, while the median values of IL-6, TGF-β, and CCL-20 tended to be higher in the case of inactive GO (**Supplementary Figure 1D**).

### Basal characteristics of peripheral blood T lymphocytes from active and inactive GO

The total numbers of CD4^+^ T lymphocytes were significantly (*p* < 0.01) higher in the peripheral blood from inactive GO than active GO (**Supplementary Figure 1E**). Furthermore, the absolute numbers of Th17 (CD4^+^IL-17^+^) and Tregs (CD4^+^Foxp3^+^) were significantly (*p* < 0.01) higher in patients with inactive GO. Although the absolute number of CD4^+^Foxp3^+^ Tregs was lower in active GO, these lymphocytes expressed significantly higher FoxP3 levels (*p* < 0.05) (**Supplementary Figure 1E**).

### Basal cytokine secretion by peripheral blood mononuclear cells from active and inactive GO

The release of cytokines associated with Tregs (transforming growth factor beta, TGF-β) and Th17 cells (IL-17) by PBMCs did not differ between active and inactive GO under basal culture conditions (**Supplementary Figure 1F**).

### Co-culture of orbital fibroblasts with autologous PBMCs

#### Effect on orbital fibroblast proliferation

**Figure 1:**
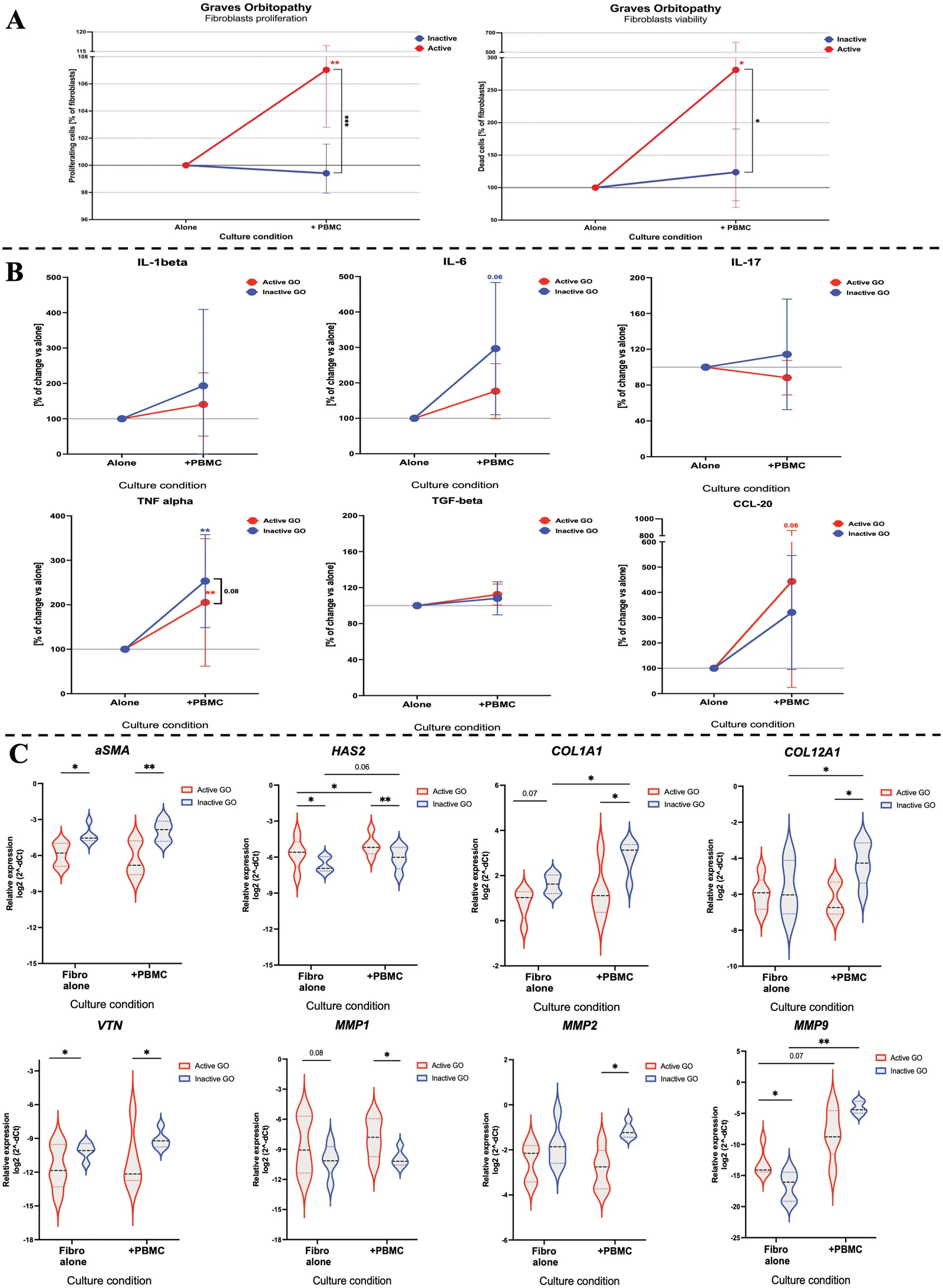
Changes in orbital fibroblast proliferation, viability, cytokine secretion, and gene expression after 48-h co-culture with matched autologous peripheral blood mononuclear cells (PBMCs). **(A)** Changes in orbital fibroblast proliferation and viability after co-culture with autologous PBMCs. **(B)** Differences in the cytokine secretion levels measured in co-culture supernatants. and **(C)** relative gene expression in orbital fibroblasts. Data are presented as median with interquartile range. Each data point represents one independent donor-derived sample (n = 5 per group). Statistical analysis was performed using a mixed-effects model followed by Fisher’s least significant difference (LSD) test. Significant differences are indicated by asterisks or exact p values (color for PBMC addition changes, with black brackets denoting differences between groups). *p < 0.05; **p < 0.01; ***p < 0.001; ****p < 0.0001.

Co-culture of the OFs from active GO with autologous PBMCs resulted in a significant increase in the OF proliferation rate, which was not observed in the case of inactive GO. Moreover, exposure of OFs to autologous PBMCs resulted in enhanced OF cell death, with significantly higher cell death in the case of active GO (**Figure 1A**).

#### Effect on cytokine secretion

Co-culture of the OFs with strictly matched autologous PBMCs enhanced the secretion of, in particular, IL-1β, IL-6, TNF-α, and CCL-20, while the effects on IL-17 and TGF-β were unnoticeable. No statistically significant differences were observed between the co-cultures from active and inactive GO (**Figure 1B**).

#### Effect on gene expression

Co-culture of OFs with PBMCs significantly (*p* = 0.03) enhanced the gene expression level of *HAS2* in active GO, while the gene expression of *COL1A1*, *COL12A1*, and *MMP-9* was significantly enhanced in the inactive GO group (*p* = 0.0241, *p* = 0.0467, and *p* = 0.0028, respectively). Moreover, co-culture had only a minimal effect on the expression of *aSMA*, *VTN*, *MMP1*, and *MMP-2*, with the difference between the fibroblasts alone persisting under co-culture conditions (**Figure 1C**).

#### Effect of methylprednisolone and vitamin D3

First, we determined the presence of *NR3C1* (glucocorticosteroid receptor) and *VDR* (vitamin D receptor) in OFs. The relative expression levels of these genes did not differ between the OFs from active GO and inactive GO (**Supplementary Figure 1B**).

The OFs from active GO showed a dose-dependent decrease in proliferation as the MP concentration increased, with significant inhibition at 1 μM MP. Co-culture with autologous PBMCs slightly altered this effect: low-dose MP (0.2 μM) mildly increased the proliferation of fibroblasts, while 1 μM MP still significantly reduced it, similar to fibroblasts cultured alone. MP also reduced fibroblast viability in a dose-dependent manner, and higher MP levels, reflecting disease activity, were associated with increased cytotoxicity in the presence of PBMCs (**Supplementary Figure 2A**).

**Figure 2:**
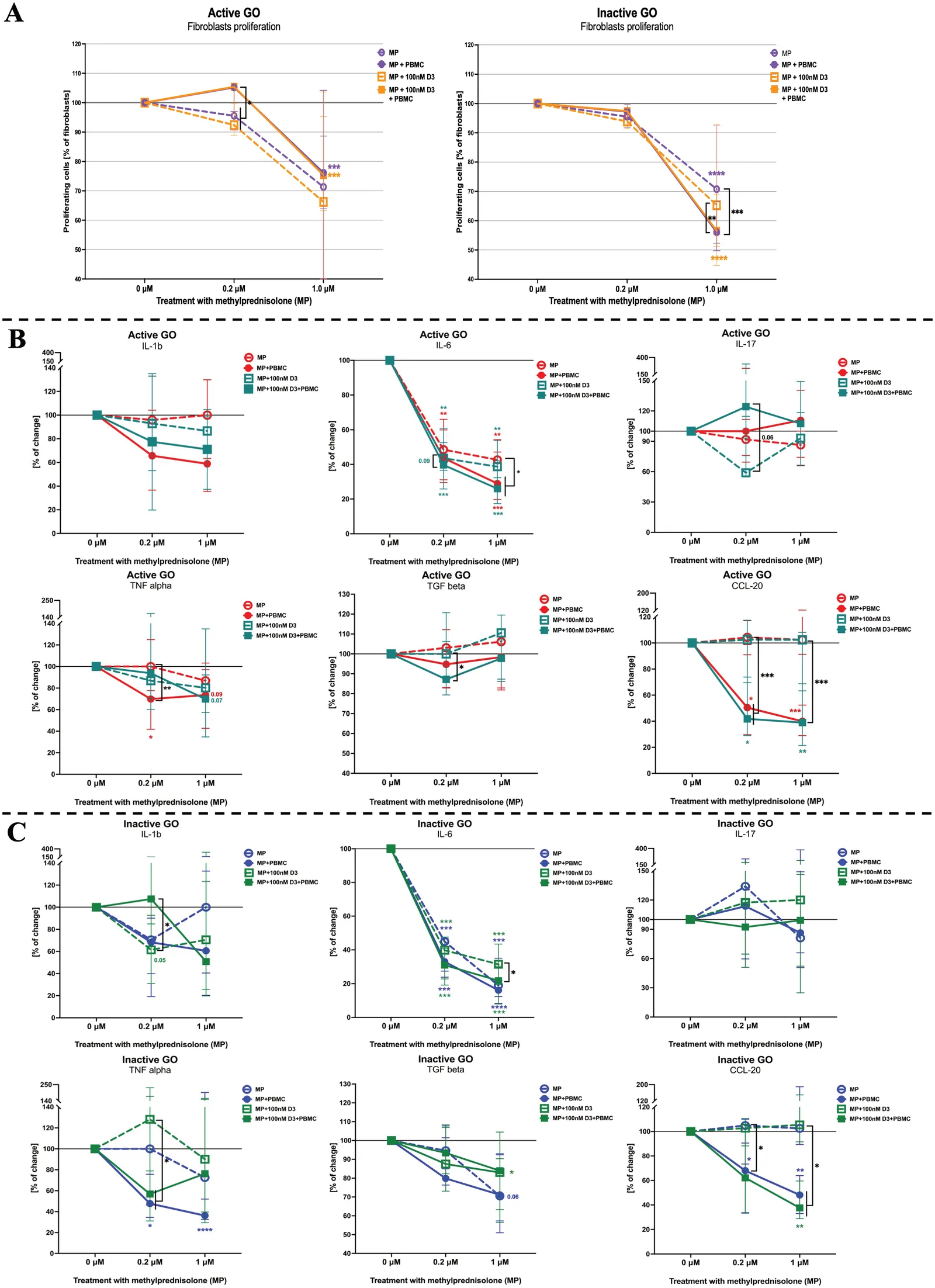
Effect of methylprednisolone (MP) and vitamin D3 (D3) on orbital fibroblast–peripheral blood mononuclear cells (PBMC) co-cultures after 48-h incubation. **(A)** Changes in orbital fibroblast proliferation in co-cultures established from patients with active and inactive Graves’ orbitopathy (GO). **(B, C)** Cytokine concentrations in co-culture supernatants from active GO **(B)** and inactive GO **(C)**. Data are presented as median with interquartile range. Each data point represents one independent donor-derived sample (n = 5 per group). Statistical analysis was performed using a mixed-effects model followed by Fisher’s least significant difference (LSD) test. Significant differences are indicated with asterisks or exact p-values (color for MP or D3 treatment changes, with black brackets denoting differences between GO groups). *p < 0.05; **p < 0.01; ***p < 0.001; ****p < 0.0001.

MP reduced the secretion of IL-6 in a concentration-dependent manner in both the active and inactive stages, regardless of the presence of PBMCs. However, the reduction was more pronounced when PBMCs were included in the culture, and this effect was slightly enhanced by the addition of vitamin D3 (**Figures 2B, C**). The TNF-α levels in inactive GO fibroblasts co-cultured with PBMCs decreased in a dose-dependent manner. The addition of vitamin D3 to MP appears to have no impact on the release of TNF-α. The secretion of TGF-β decreased dose-dependently only in inactive GO, while the CCL20 levels dropped significantly in the PBMC co-cultures in both groups, independent of vitamin D3. This reduction was more pronounced in the active GO group, even at low MP doses (**Figures 2B, C**).

#### Effect of methylprednisolone and vitamin D3 on the expression of the remodeling-related genes in orbital fibroblasts

**Figure 3:**
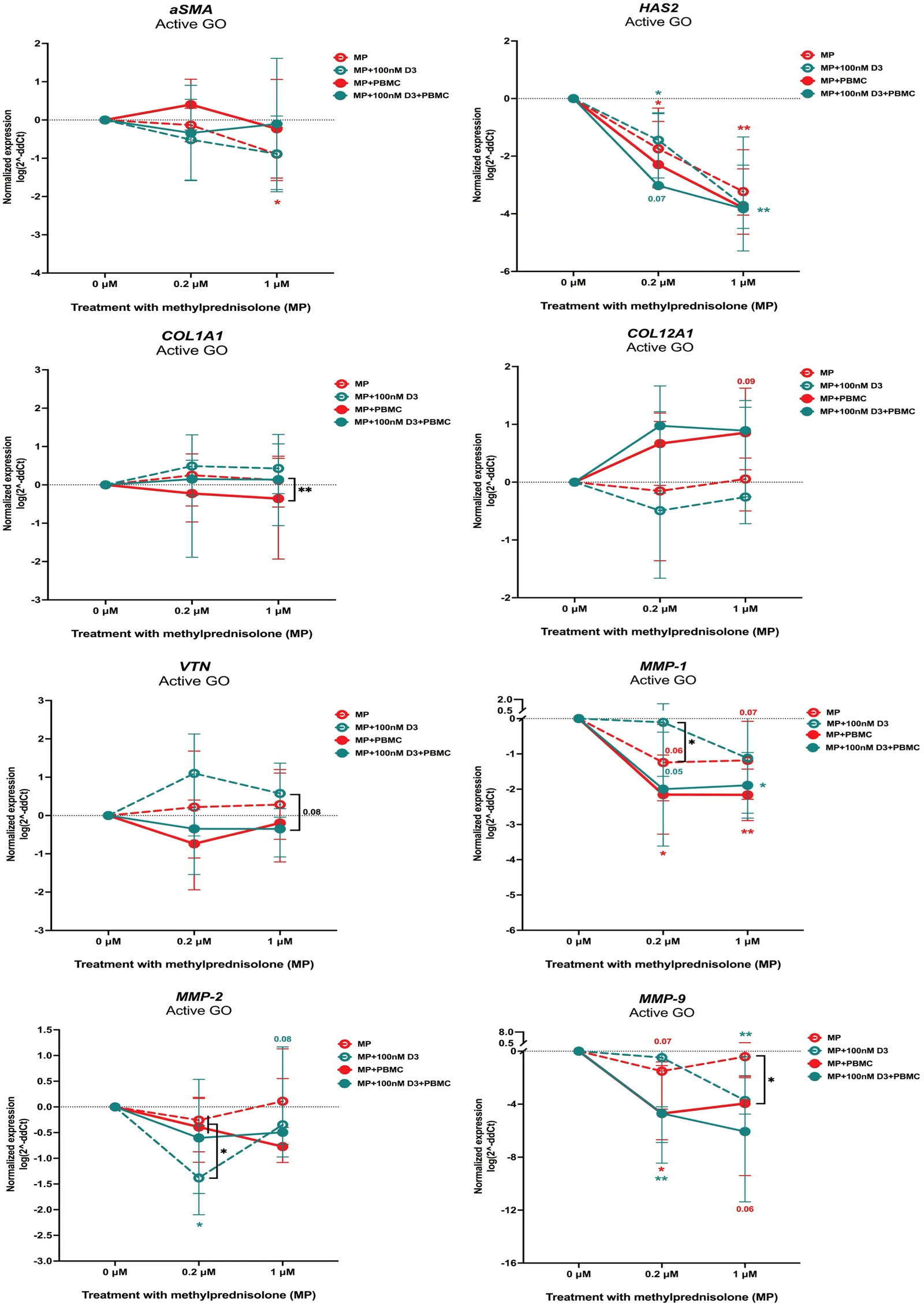
Effect of methylprednisolone (MP) and vitamin D3 (D3) on the expression of the remodeling- and inflammation-related genes in orbital fibroblasts from patients with active Graves’ orbitopathy (GO) after 48-h co-culture with matched autologous peripheral blood mononuclear cells (PBMCs). Data are presented as median with interquartile range. Each data point represents one independent donor-derived sample (*n* = 5). Statistical analysis was performed using a mixed-effects model followed by Fisher’s least significant difference (LSD) test. *Colored asterisks* or exact *p*-values indicate differences among the studied steroid concentrations, whereas *black brackets* indicate comparisons between experimental groups. Statistical significance was defined as color*p < 0.05; **p < 0.01; ***p < 0.001; ****p < 0.0001.

**Figure 4:**
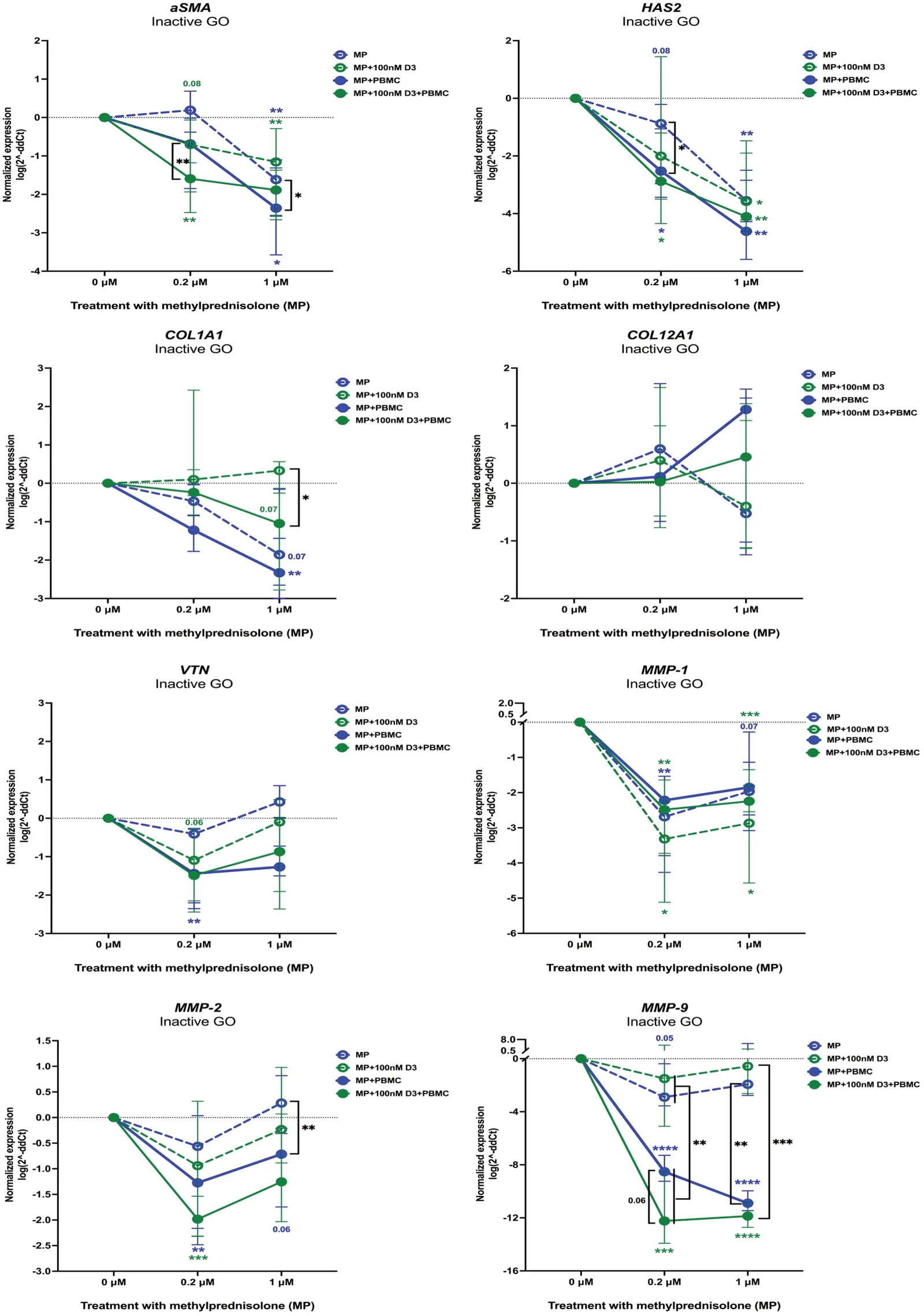
Effect of methylprednisolone (MP) and vitamin D3 (D3) on the expression of the remodeling- and inflammation-related genes in orbital fibroblasts from patients with inactive Graves’ orbitopathy (GO) after 48-h co-culture with matched autologous peripheral blood mononuclear cells (PBMCs). Data are presented as median with interquartile range. Each data point represents one independent donor-derived sample (*n* = 5). Statistical analysis was performed using a mixed-effects model followed by Fisher’s least significant difference (LSD) test. *Colored asterisks* or exact *p*-values indicate differences among the studied steroid concentrations, whereas *black brackets* indicate comparisons between experimental groups. Statistical significance was defined as color*p < 0.05; **p < 0.01; ***p < 0.001; ****p < 0.0001.

Only the 1 μM of MP decreased the expression of *aSMA* in active fibroblasts (**Figure 3**). Moreover, in inactive GO fibroblasts, co-culture with PBMCs enhanced the reduction of *aSMA* expression regardless of the presence of vitamin D3 (**Figure 4**). Notably, the addition of MP was associated with a decline in *HAS2* expression in a dose-dependent manner. The addition of PBMCs enhanced this reduction of *HAS2* expression. Co-cultured inactive fibroblasts showed a decrease in *COL12A1* expression with increasing MP concentration (**Figures 3**, **4**).

The expression of *MMP1* decreased following MP treatment, with the strongest response observed in active fibroblasts co-cultured with PBMCs. The addition of vitamin D3 did not enhance the effect of MP in either fibroblasts from active or inactive disease. Notably, the most substantial reduction in *MMP1* was observed in inactive fibroblasts, regardless of the culture conditions (**Figures 3**, **4**). Alterations in the *MMP2* expression were most prominent in inactive fibroblasts. Culturing of these cells, with or without PBMCs, resulted in a reduced *MMP2* expression at low MP doses. This effect was significantly amplified by the addition of vitamin D3 (**Figure 4**).

The expression of *MMP-9* was significantly decreased in co-cultured fibroblasts with the addition of MP, and more pronounced changes were observed in the fibroblasts from inactive disease. In the fibroblasts from both active and inactive disease, the addition of vitamin D3 to co-cultures enhanced the effect of MP in a dose-dependent manner (**Figures 3**, **4**).

## Discussion

Chronic tissue inflammation is often characterized by the accumulation and excessive activity of fibroblasts, with a key role in tissue healing and remodeling (29). Our study revealed that the OF activity in patients with GO varies with the disease stage (active *versus* inactive) and is influenced by immune cells. Furthermore, the effects of MP and vitamin D3 were found to be related to the disease activity.

Assessment of the basal production of a selection of cytokines known to be involved in GO showed no significant differences between the OFs and PBMCs obtained from patients with active or inactive GO. Moreover, the numbers of Tregs were reduced within the PBMC fraction from patients with active GO, supporting the inflammatory condition of the periorbital tissue. Liu et al. identified an increase in systemic inflammation in GO, driven by cytotoxic T-cell activation and the shift of Tregs toward a pro-inflammatory phenotype. This transition might result in the infiltration of Tregs into periorbital tissues and promote fibrosis (30).

Th17-related fibrosis has been linked to fibroproliferative diseases (31), including GO, in which elevated Th17 cells and IL-17A levels were observed, along with the infiltration of IL-17A-producing cells into orbital tissue. IL-17A stimulates the production of inflammatory and ECM molecules by OFs, without affecting apoptosis or necrosis (32, 33). In contrast, our results did not reveal significantly elevated IL-17 secretion by either OFs or PBMCs from active GO. This may be related to both the limited sample size and methodological differences, as previous studies assessed IL-17 in tissue sections or plasma using ELISA, while our data were derived from *in vitro* cell culture supernatants.

Establishing the precise mechanisms underlying the active and inactive phases of GO requires identifying multiple molecular networks, including those derived from immune cells and those associated with soft connective tissue. Virakul et al. identified the overexpression of proteins associated with inflammation, proliferation, adipogenesis, and hyaluronan deposition in the OFs from active GO. In contrast, the OFs from inactive GO were predominantly linked to fibrosis and ECM remodeling (34). Our study also revealed that the OFs from active GO exhibited lower expression of the fibrosis-related (*aSMA*) and ECM-related (*VTN*) genes. It is noteworthy that *aSMA* is closely associated with the TGF-β pathway, a key pro-fibrotic molecular pathway (35). Simultaneously, we observed that the OFs from active GO exhibited elevated levels of the enzymes involved in ECM degradation, particularly MMP-9. Our findings align with previously published data indicating increased levels of serum MMP-9 in active GO (36). Collectively, these findings emphasize the critical role of OFs in shaping the inflammatory microenvironment within the periorbital tissue during the active disease phase of GO.

GO involves complex cellular and humoral factors. Here, we found that interactions between PBMCs and OFs significantly promote fibroblast expansion. These effects extended further towards the modulation of cytokine release by fibroblasts. The presence of PBMCs was associated with increased TNF-α production, particularly in cells from inactive GO. Lu et al. reported that stimulation with bovine TSH significantly affects monocyte-derived fibrocytes, increasing TNF-α expression, consistent with our findings (37). This phenomenon may be associated with immune exhaustion, as indicated by the lack of responsiveness in the co-cultured cells during the active state and the markedly elevated levels of IL-6 observed in the inactive GO group, in contrast. Conversely, we found that active GO was associated with a modest increase in the CCL-20 levels during fibroblast–PBMC interaction compared with the samples from inactive subjects. The interactions between fibroblasts and PBMCs influenced the expression of the genes involved in tissue remodeling and fibrosis. Autologous PBMCs notably increased the expression of MMP*-9* and *HAS2*, with the latter contributing to an early change in hyaluronan deposition and to processes such as cell motility and wound healing, which are both physiological and pathological (38). PBMCs also upregulated the ECM-related genes (e.g., *COL1A1* and *COL12A1*) and *MMP-9*, which may help regulate the ECM structure (39). While PBMCs did not cause major changes between the active and inactive co-cultures individually, differences emerged in the expression of *αSMA* and *VTN*, with the elevation of *VTN* likely driven by pro-inflammatory cytokines. This aligns with the findings by Scaffidi et al., which linked *VTN* to fibroblast activation and ECM remodeling under inflammatory conditions (40).

Treatment with MP significantly reduced the proliferation of the OFs from both active and inactive disease, which displayed equal expression of *GCR* and *VDR*. In addition, the *VDR* gene variants showed no clear link to the occurrence or severity of thyroid-associated ophthalmopathy (41). Interestingly, in inactive GO, the presence of matched autologous PBMCs in co-culture was associated with an intensified anti-proliferative response to MP, an effect not observed in active disease. Within each study group, the involvement of PBMCs in the experimental setting correlated with a higher frequency of dead cells when MP, with or without vitamin D3, was administered. This phenomenon was present at the highest MP concentration in the inactive group, but even at a fivefold reduction in active GO. Our results are in contrast to those of Galgoczi et al., who reported that the application of steroids (e.g., hydrocortisone, prednisolone, MP, or dexamethasone) did not affect the proliferation of OFs (42). MP treatment in the fibroblast–PBMC co-cultures significantly reduced the pro-inflammatory cytokine secretion in a dose-dependent manner. The combination of MP and vitamin D3 most effectively lowered the IL-6 levels, in particular in active GO with autologous PBMCs. The TNF-α and TGF-β levels also decreased, although the latter only in inactive GO, while CCL-20 was notably reduced in active GO even at low doses. As CCL-20 is involved in immune cell recruitment and inflammation (43, 44), its suppression aligns with reduced cell proliferation. Vitamin D3 may enhance steroid efficacy by boosting anti-inflammatory effects, which we have previously described (45, 46).

Variations in the pro-remodeling- and fibrosis-related gene expression may be modulated by steroids, with or without vitamin D3. In PBMC co-cultures, only the inactive GO fibroblasts showed reduced *αSMA*, even at low MP doses, while the expression of *HAS2* declined in both groups, supporting previous findings on steroid-mediated hyaluronan inhibition (42). Active GO fibroblasts showed increased *COL12A1*, while *COL1A1* and *VTN* decreased in the samples from inactive GO. Current reports suggest that immune-mediated signaling, primarily through cytokine secretion, allows PBMCs to influence collagen deposition in fibroblasts. Although TGF-β and TNF-α are known to promote collagen synthesis (47, 48), we observed no significant changes in the levels of these cytokines secreted by PBMCs, suggesting that these effects result from the interactions between PBMCs and fibroblasts.

In reference to metalloproteinases, a significant reduction of *MMP-1* in active GO was observed exclusively in the presence of autologous PBMCs. Interestingly, *MMP-2* declined only in active fibroblasts treated with the lowest MP concentration, whereas in the inactive group, the addition of PBMCs was required to achieve comparable effects. The PBMC environment was also essential in establishing a reduction of *MMP-9* by MP. In fibroblasts, vitamin D3 was necessary alongside MP to achieve a decrease in *MMP-9* in active GO cells. Similar observations were made by Halder et al., who found that vitamin D3 reduced the expression of MMP-2 and MMP-9 in a fibroid cell line (49). The changes we observed in the fibrosis- and early remodeling-associated genes were GO stage-dependent. Accordingly, the data suggest that, in the majority of cases, MP exhibits a lower ability to modulate profibrotic processes and the ECM elements in fibroblasts from active GO patients than in those from the inactive group.

Our study has several limitations. The sample size reflects the limited number of paired OFs and autologous PBMCs and warrants validation in larger cohorts. The use of total PBMCs enabled mutual immunological interactions rather than the identification of specific immune cell subsets, thereby requiring further experiments. In addition, the experiments were performed at a single time point, providing only a snapshot of interactions. Finally, the use of exogenous modulators (MP and vitamin D3) adds complexity, and their effects should be interpreted as preliminary and modulatory. Another limitation of this study is that the expression of the remodeling- and fibrosis-related markers was assessed only at the transcript level. Consequently, future studies incorporating protein-level analyses and direct assessment of the ECM components, such as hyaluronan, are warranted to validate the functional significance of the observed transcriptional changes.

## Conclusion

Orbital immune infiltration in GO may be a key factor exacerbating the inflammatory process. Building upon previous evidence demonstrating interactions between autologous immune cells and OFs, our study further characterizes the crosstalk between matched autologous PBMCs and OFs by evaluating fibroblast proliferation, immune responses, cytokine secretion, and remodeling-related gene expression. Fibroblasts from inactive GO showed increased pro-remodeling-related gene expression (*αSMA* and *VTN*), while those from active GO expressed more pro-inflammatory genes. Co-culture with autologous PBMCs activated fibroblasts, enhancing cytokine release and altering ECM profiles, highlighting the importance of cell–cell interactions in disease progression. Importantly, MP reduced fibroblast proliferation, particularly in active GO, and downregulated the early remodeling-associated (including ECM-related) gene expression in inactive GO fibroblasts. Finally, MP, combined with vitamin D3, further suppressed IL-6 and CCL-20 secretion, suggesting that vitamin D3 is of therapeutic interest for reducing orbital inflammation in GO.

## Data Availability

The raw data supporting the conclusions of this article will be made available by the authors, without undue reservation.

## References

[1] Antonelli A Fallahi P Elia G Ragusa F Paparo SR Ruffilli I . Graves' disease: clinical manifestations, immune pathogenesis (cytokines and chemokines) and therapy. Best Pract Res Clin Endocrinol Metab. (2020) 34:101388. doi: 10.1016/j.beem.2020.101388 32059832

[2] Smith TJ Hegedüs L . Graves' disease. N Engl J Med. (2016) 375:1552–65. doi: 10.1056/NEJMra1510030 27797318

[3] Bahn RS . Graves' ophthalmopathy. N Engl J Med. (2010) 362:726–38. doi: 10.1056/nejmra0905750 20181974 PMC3902010

[4] Bartalena L Kahaly GJ Baldeschi L Dayan CM Eckstein A Marcocci C . The 2021 European Group on Graves' orbitopathy (EUGOGO) clinical practice guidelines for the medical management of Graves' orbitopathy. Eur J Endocrinol. (2021) 185:G43–67. doi: 10.1530/eje-21-0479 34297684

[5] Longo CM Higgins PJ . Molecular biomarkers of Graves' ophthalmopathy. Exp Mol Pathol. (2018) 106:1–6. doi: 10.1016/j.yexmp.2018.11.004 30414981 PMC6381289

[6] Wiersinga WM Bartalena L . Epidemiology and prevention of Graves' ophthalmopathy. Thyroid. (2002) 12:855–60. doi: 10.1089/105072502761016476 12487767

[7] Khong JJ McNab AA Ebeling PR Craig JE Selva D . Pathogenesis of thyroid eye disease: review and update on molecular mechanisms. Br J Ophthalmol. (2016) 100:142–50. doi: 10.1136/bjophthalmol-2015-307399 26567024

[8] Cui X Wang F Liu C . A review of TSHR- and IGF-1R-related pathogenesis and treatment of Graves' orbitopathy. Front Immunol. (2023) 14:1062045. doi: 10.3389/fimmu.2023.1062045 36742308 PMC9893276

[9] Furmaniak J Sanders J Sanders P Li Y Rees Smith B . TSH receptor specific monoclonal autoantibody K1-70. Clin Endocrinol (Oxf). (2022) 96:878–87. doi: 10.1111/cen.14681 35088429 PMC9305464

[10] Dik WA Virakul S van Steensel L . Current perspectives on the role of orbital fibroblasts in the pathogenesis of Graves' ophthalmopathy. Exp Eye Res. (2016) 142:83–91. doi: 10.1016/j.exer.2015.02.007 26675405

[11] Feldon SE Park DJ O'Loughlin CW Nguyen VT Landskroner-Eiger S Chang D . Autologous T-lymphocytes stimulate proliferation of orbital fibroblasts derived from patients with Graves' ophthalmopathy. Invest Ophthalmol Vis Sci. (2005) 46:3913–21. doi: 10.1167/iovs.05-0605 16249464

[12] Wang ZM Wang ZY Lu Y . The role of cell mediated immunopathogenesis in thyroid-associated ophthalmopathy. Int J Ophthalmol. (2019) 12:1209–14. doi: 10.18240/ijo.2019.07.24 31341815 PMC6629801

[13] Huang Y Fang S Li D Zhou H Li B Fan X . The involvement of T cell pathogenesis in thyroid-associated ophthalmopathy. Eye (Lond). (2019) 33:176–82. doi: 10.1038/s41433-018-0279-9 30531993 PMC6367411

[14] Fang S Lu Y Huang Y Zhou H Fan X . Mechanisms that underly T cell immunity in Graves' orbitopathy. Front Endocrinol (Lausanne). (2021) 12:648732. doi: 10.3389/fendo.2021.648732 33868176 PMC8049604

[15] Fang S Huang Y Wang N Zhang S Zhong S Li Y . Insights into local orbital immunity: evidence for the involvement of the Th17 cell pathway in thyroid-associated ophthalmopathy. J Clin Endocrinol Metab. (2019) 104:1697–711. doi: 10.1210/jc.2018-01626 30517642

[16] Eckstein AK Quadbeck B Tews S Mann K Krüger C Mohr CH . Thyroid associated ophthalmopathy: evidence for CD4(+) gammadelta T cells; de novo differentiation of RFD7(+) macrophages, but not of RFD1(+) dendritic cells; and loss of gammadelta and alphabeta T cell receptor expression. Br J Ophthalmol. (2004) 88:803–8. doi: 10.1136/bjo.2003.035915 15148216 PMC1772193

[17] Fang S Zhang S Huang Y Wu Y Lu Y Zhong S . Evidence for associations between Th1/Th17 "hybrid" phenotype and altered lipometabolism in very severe Graves' orbitopathy. J Clin Endocrinol Metab. (2020) 6(2020):dgaa124. doi: 10.1210/clinem/dgaa124 32173759

[18] Wang Y Chen Z Wang T Guo H Liu Y Dang N . A novel CD4+ CTL subtype characterized by chemotaxis and inflammation is involved in the pathogenesis of Graves' orbitopathy. Cell Mol Immunol. (2021) 18:735–45. doi: 10.1038/s41423-020-00615-2 33514849 PMC8027210

[19] Görtz GE Philipp S Bruderek K Jesenek C Horstmann M Henning Y . Macrophage-orbital fibroblast interaction and hypoxia promote inflammation and adipogenesis in Graves' orbitopathy. Endocrinology. (2022) 64(2):bqac203. doi: 10.1210/endocr/bqac203 36477465

[20] Zhang P Zhu H . Cytokines in thyroid-associated ophthalmopathy. J Immunol Res. (2022) 2022:2528046. doi: 10.1155/2022/2528046 36419958 PMC9678454

[21] Gianoukakis AG Khadavi N Smith TJ . Cytokines, Graves' disease, and thyroid-associated ophthalmopathy. Thyroid. (2008) 18:953–8. doi: 10.1089/thy.2007.0405 18713026 PMC2879490

[22] Ferrari SM Paparo SR Ragusa F Elia G Mazzi V Patrizio A . Chemokines in thyroid autoimmunity. Best Pract Res Clin Endocrinol Metab. (2023) 37:101773. doi: 10.1016/j.beem.2023.101773 36907786

[23] Zhao R Zhang W Ma C Zhao Y Xiong R Wang H . Immunomodulatory function of vitamin D and its role in autoimmune thyroid disease. Front Immunol. (2021) 12:574967. doi: 10.3389/fimmu.2021.574967 33679732 PMC7933459

[24] Gallo D Bruno A Gallazzi M Cattaneo SAM Veronesi G Genoni A . Immunomodulatory role of vitamin D and selenium supplementation in newly diagnosed Graves' disease patients during methimazole treatment. Front Endocrinol (Lausanne). (2023) 14:1145811. doi: 10.3389/fendo.2023.1145811 37124743 PMC10141462

[25] Mele C Caputo M Bisceglia A Samà MT Zavattaro M Aimaretti G . Immunomodulatory effects of vitamin D in thyroid diseases. Nutrients. (2020) 12(5):1444. doi: 10.3390/nu12051444 32429416 PMC7284826

[26] Sawicka-Gutaj N Gruszczyński D Zawalna N Nijakowski K Skiba A Pochylski M . Safety of non-standard regimen of systemic steroid therapy in patients with Graves' orbitopathy: a single-centre experience. Pharmacol Rep. (2024) 76:185–94. doi: 10.1007/s43440-023-00567-0 38273183 PMC10830746

[27] Walasik-Szemplińska D Kamiński G Sudoł-Szopińska I . Life-threatening complications of high doses of intravenous methylprednisolone for treatment of Graves' orbitopathy. Thyroid Res. (2019) 12:13. doi: 10.1186/s13044-019-0074-0 31890036 PMC6927113

[28] van Steensel L Paridaens D Schrijver B Dingjan GM van Daele PL van Hagen PM . Imatinib mesylate and AMN107 inhibit PDGF-signaling in orbital fibroblasts: a potential treatment for Graves' ophthalmopathy. Invest Ophthalmol Vis Sci. (2009) 50:3091–8. doi: 10.1167/iovs.08-2443 19234339

[29] Kendall RT Feghali-Bostwick CA . Fibroblasts in fibrosis: novel roles and mediators. Front Pharmacol. (2014) 5:123. doi: 10.3389/fphar.2014.00123 24904424 PMC4034148

[30] Liu Z Ke SR Shi ZX Zhou M Sun L Sun QH . Dynamic transition of Tregs to cytotoxic phenotype amid systemic inflammation in Graves' ophthalmopathy. JCI Insight. (2024) 9(22):e181488. doi: 10.1172/jci.insight.181488 39365735 PMC11601897

[31] Wynn TA Ramalingam TR . Mechanisms of fibrosis: therapeutic translation for fibrotic disease. Nat Med. (2012) 18:1028–40. doi: 10.1038/nm.2807 22772564 PMC3405917

[32] Fang S Huang Y Wang S Zhang Y Luo X Liu L . IL-17A exacerbates fibrosis by promoting the proinflammatory and profibrotic function of orbital fibroblasts in TAO. J Clin Endocrinol Metab. (2016) 101:2955–65. doi: 10.1210/jc.2016-1882 27224264

[33] Fang S Huang Y Zhong S Zhang Y Liu X Wang Y . IL-17A promotes RANTES expression, but not IL-16, in orbital fibroblasts via CD40-CD40L combination in thyroid-associated ophthalmopathy. Invest Ophthalmol Vis Sci. (2016) 57:6123–33. doi: 10.1167/iovs.16-20199 27832278

[34] Virakul S Somparn P Pisitkun T van der Spek PJ Dalm VASH Paridaens D . Integrative analysis of proteomics and DNA methylation in orbital fibroblasts from Graves' ophthalmopathy. Front Endocrinol (Lausanne). (2020) 11:619989. doi: 10.3389/fendo.2020.619989 33658982 PMC7919747

[35] Morishige N Murata S Nakamura Y Azumi H Shin-Gyou-Uchi R Oki KT . Coordinated regulation of palladin and α-smooth muscle actin by transforming growth factor-β in human corneal fibroblasts. Invest Ophthalmol Vis Sci. (2016) 57:3360–8. doi: 10.1167/iovs.15-18763 27367503

[36] Riguetto CM Barbosa EB Atihe CC Reis F Alves M Zantut-Wittmann DE . Interaction of MMP-9 in the active phase of Graves' disease with and without ophthalmopathy. Am J Physiol Endocrinol Metab. (2024) 327:E577–84. doi: 10.1152/ajpendo.00166.2024 39259164 PMC11482230

[37] Lu Y Atkins SJ Fernando R Trierweiler A Mester T Grisolia ABD . CD34- orbital fibroblasts from patients with thyroid-associated ophthalmopathy modulate TNF-α expression in CD34+ fibroblasts and fibrocytes. Invest Ophthalmol Vis Sci. (2018) 59:2615–22. doi: 10.1167/iovs.18-23951 29847668 PMC5968835

[38] Amornsupak K Jamjuntra P Warnnissorn M O-Charoenrat P Sa-Nguanraksa D Thuwajit P . High ASMA. Clin Breast Cancer. (2017) 17:441–452.e2. doi: 10.1016/j.clbc.2017.04.007 28533055

[39] Ohbayashi H Shimokata K . Matrix metalloproteinase-9 and airway remodeling in asthma. Curr Drug Targets Inflammation Allergy. (2005) 4:177–81. doi: 10.2174/1568010053586246 15853739

[40] Scaffidi AK Moodley YP Weichselbaum M Thompson PJ Knight DA . Regulation of human lung fibroblast phenotype and function by vitronectin and vitronectin integrins. J Cell Sci. (2001) 114:3507–16. doi: 10.1242/jcs.114.19.3507 11682610

[41] Maciejewski A Kowalczyk MJ Gasińska T Szeliga A Prendecki M Dorszewska J . The role of vitamin D receptor gene polymorphisms in thyroid-associated orbitopathy. Ocul Immunol Inflammation. (2020) 28:354–61. doi: 10.1080/09273948.2019.1629605 31424978

[42] Galgoczi E Katko M Papp FR Csiki R Csiha S Erdei A . Glucocorticoids directly affect hyaluronan production of orbital fibroblasts; a potential pleiotropic effect in Graves' orbitopathy. Molecules. (2022) 28(1):15. doi: 10.3390/molecules28010015 36615214 PMC9822010

[43] Kadomoto S Izumi K Mizokami A . The CCL20-CCR6 axis in cancer progression. Int J Mol Sci. (2020) 21(15):5186. doi: 10.3390/ijms21155186 32707869 PMC7432448

[44] Ekperikpe US Poudel B Shields CA Mandal S Cornelius DC Williams JM . Neutralizing MIP3. J Pharmacol Exp Ther. (2023) 384:445–54. doi: 10.1124/jpet.122.001298 36507846 PMC9976792

[45] Zhang Y Leung DY Goleva E . Anti-inflammatory and corticosteroid-enhancing actions of vitamin D in monocytes of patients with steroid-resistant and those with steroid-sensitive asthma. J Allergy Clin Immunol. (2014) 133:1744–1752.e1. doi: 10.1016/j.jaci.2013.12.004 24418482 PMC4040328

[46] Grubczak K Lipinska D Eljaszewicz A Singh P Radzikowska U Miklasz P . Vitamin D3 treatment decreases frequencies of CD16-positive and TNF-α-secreting monocytes in asthmatic patients. Int Arch Allergy Immunol. (2015) 166:170–6. doi: 10.1159/000380882 25872112

[47] Dufour AM Alvarez M Russo B Chizzolini C . Interleukin-6 and type-I collagen production by systemic sclerosis fibroblasts are differentially regulated by interleukin-17A in the presence of transforming growth factor-beta 1. Front Immunol. (2018) 9:1865. doi: 10.3389/fimmu.2018.01865 30150989 PMC6099180

[48] Szondy Z Pallai A . Transmembrane TNF-alpha reverse signaling leading to TGF-beta production is selectively activated by TNF targeting molecules: therapeutic implications. Pharmacol Res. (2017) 115:124–32. doi: 10.1016/j.phrs.2016.11.025 27888159

[49] Halder SK Osteen KG Al-Hendy A . Vitamin D3 inhibits expression and activities of matrix metalloproteinase-2 and -9 in human uterine fibroid cells. Hum Reprod. (2013) 28:2407–16. doi: 10.1093/humrep/det265 23814095 PMC3748859

