# Supplementary material for: In vitro evaluation of the interaction between orbital fibroblasts and PBMCs from Graves’ orbitopathy patients in triggering periorbital remodeling
Source: Front Immunol. 2026 Sep 9;17:1728194. doi: 10.3389/fimmu.2026.1728194 (PMC13597260; doi:10.3389/fimmu.2026.1728194)
Supplement: Supplementary file 1 [file DataSheet1.pdf]

Supplementary Material

***In vitro* evaluation of the interaction between orbital fibroblasts and peripheral blood mononuclear cells from Graves' orbitopathy patients in triggering periorbital remodeling.**

Aleksandra Starosz[1,*], Willem A. Dik[2,3], Dion Paridaens[4,5], J. Conny PA van Holten Neelen[2], Stéphanie Goes[4], Przemysław Pawłowski[6], Marcin Moniuszko[1,7], Kamil Grubczak[1*]

Supplementary Figures

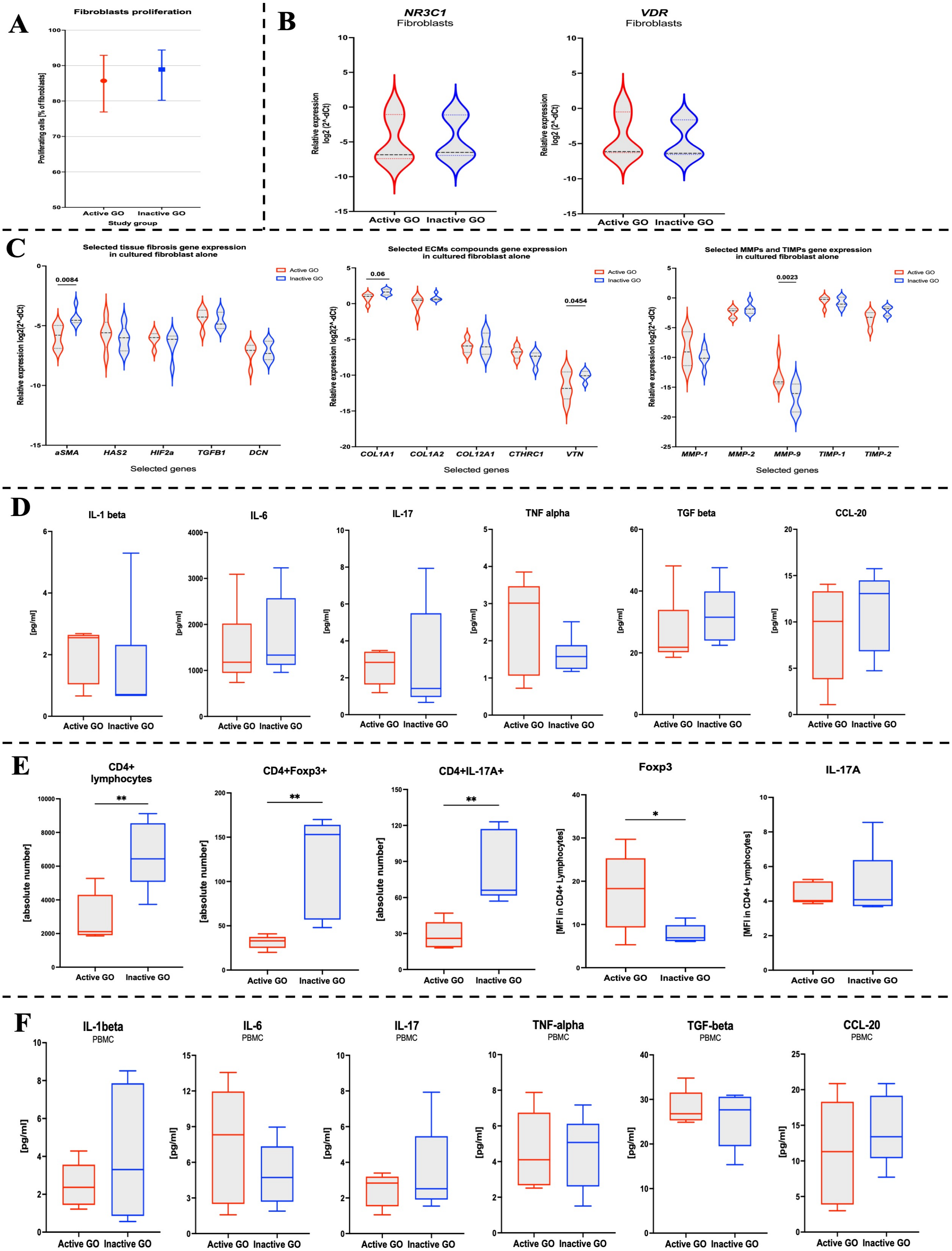
A
Fibroblasts proliferation
Proliferating cells [% of fibroblasts]
Active GO
Inactive GO
Study group
B
NR3C1
Fibroblasts
VDR
Fibroblasts
Relative expression
log2 (2^-dCt)
Active GO
Inactive GO
C
Selected tissue fibrosis gene expression
in cultured fibroblast alone
0.0084
aSMA
HAS2
HIF2a
TGFB1
DCN
Selected genes
Selected ECMs compounds gene expression
in cultured fibroblast alone
0.06
0.0454
COL1A1
COL1A2
COL12A1
CTHRC1
VTN
Selected MMPs and TIMPs gene expression
in cultured fibroblast alone
0.0023
MMP-1
MMP-2
MMP-9
TIMP-1
TIMP-2
Relative expression log2(2^-dCt)
D
IL-1 beta
IL-6
IL-17
TNF alpha
TGF beta
CCL-20
[pg/ml]
E
CD4+
lymphocytes
CD4+Foxp3+
CD4+IL-17A+
Foxp3
IL-17A
[absolute number]
[MFI in CD4+ Lymphocytes]
F
IL-1beta
IL-6
IL-17
TNF-alpha
TGF-beta
CCL-20
PBMC
[pg/ml]

Supplementary Figure 1. Baseline characteristics of orbital fibroblasts from active and inactive orbitopathy patients. Proliferation rate of the active and inactive fibroblasts (A). Baseline expression of glucocorticoid receptors (*NR3C1*) and vitamin D3 receptor (*VDR*) genes on active and inactive orbitopathy fibroblasts (B). Expression of genes related to ECM (C). Cytokine secretion by orbital fibroblasts (D). Phenotype of blood autologous lymphocytes (absolute numbers and Foxp3/IL-17A MFI) (E). Cytokines released by PBMCs. (F). Significant results indicated with asterisks or exact p values: * p<0.05; ** p<0.01; *** p<0.001; **** p<0.0001.

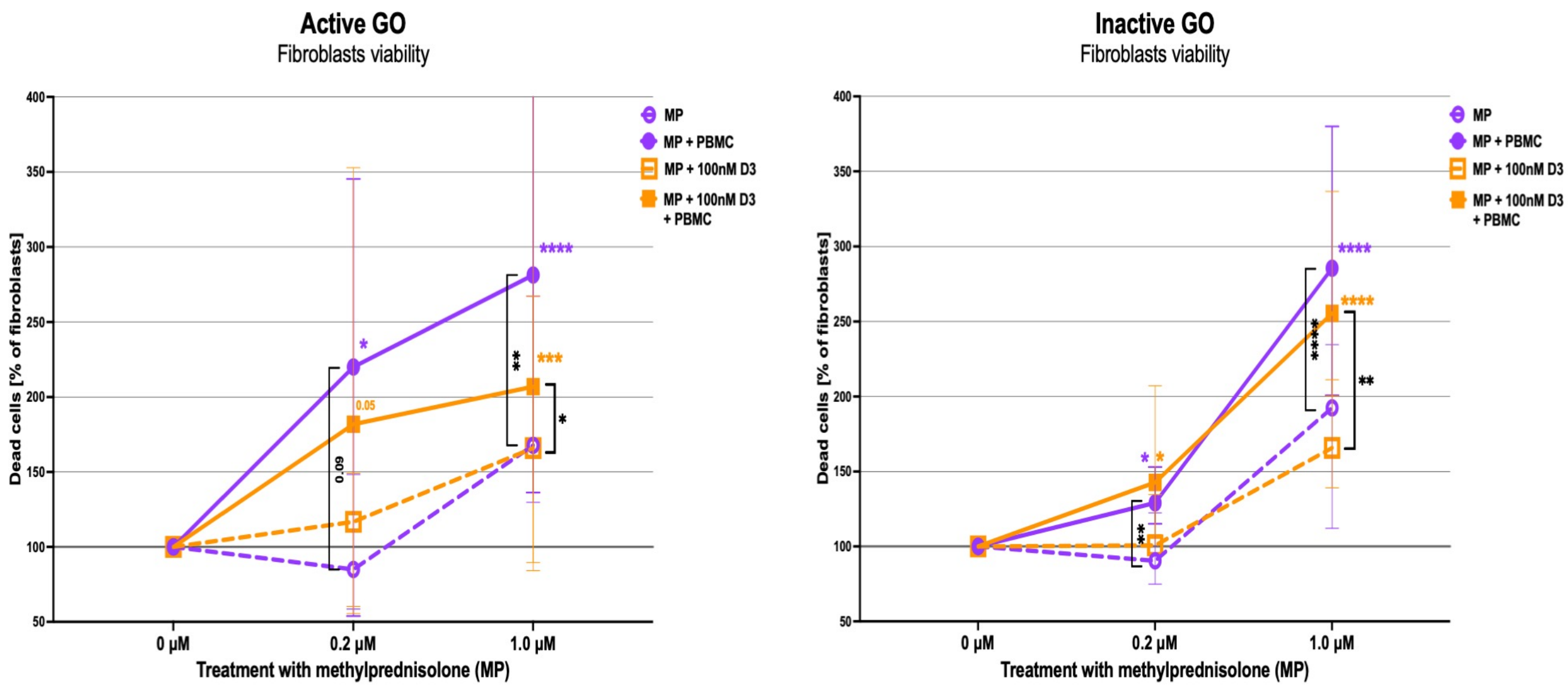

Supplementary Figure 2. Changes in the orbital fibroblasts viability after 48-hour co-culture with autologous PBMC, with/without methylprednisolone (MP) and vitamin D3 (D3). Significant differences were distinguished with asterisks or exact p values (color for MP or D3 addition changes; black brackets for differences between groups): * p<0.05; ** p<0.01; *** p<0.001; **** p<0.0001.

Supplementary Tables

Supplementary Table 1.

A.

**Group**	**Patients' ID**	**Age [years]**	**Gender**	**Exophthalmos [mm]**
Active GO	GO-49	-	Male	-
Active GO	GO-90	66	Female	18 - 22
Active GO	GO-37	78	Female	22,5 - 21,5
Active GO	GO-222	57	Male	27 - 29
Active GO	GO-215	54	Female	27 - 28
Inactive GO	GO-42	48	Female	25 - 26
Inactive GO	GO-52	26	Female	18,5 - 14,5
Inactive GO	GO-53	42	Female	16 - 18
Inactive GO	GO-55	28	Female	21 - 22
Inactive GO	GO-89	31	Female	20 - 21

B.

	Active GO (n = 5)	Inactive GO (n = 5)
**Mean Age [years]**	64 [54-78]	35 [28-48]
**Disease onset mean age [years]**	59 [54-66]	33 [23-46]
**Gender (M/F)**	2/3	0/5
**Clinical Activity Score (CAS) range**	5-7	0
**Smoking History (yes/no)**	3/0	2/2
**Euthyroid (yes/no)**	3/0	3/0

Supplementary Table 1. Characteristics of patients with active and inactive Graves' orbitopathy whose tissue samples and peripheral blood mononuclear cells were included in the study: sample coding (A) and clinical features (B).

Supplementary Table 2.

Gene name	Assay ID	Biological process
EEF1A1	Hs05015684_g1	housekeeping gene
ACTA2	Hs00426835_g1	cytoskeleton organization, fibrosis
HAS2	Hs00193435_m1	ECM synthesis
HIF2a	Hs01026149_m1	hypoxia response
TGFB1	Hs00998133_m1	fibrosis, inflammation, immune regulation
DCN	Hs00754870_s1	ECM organization
COL1A1	Hs00164004_m1	ECM composition, fibrosis
COL1A2	Hs01028956_m1	ECM composition, tissue remodeling
COL12A1	Hs00189184_m1	ECM structural integrity
CTHRC1	Hs00298917_m1	Tissue remodeling, cell migration
VTN	Hs00169863_m1	ECM remodeling, cell adhesion
MMP1	Hs00899658_m1	ECM degradation, inflammation
MMP2	Hs01548727_m1	ECM degradation, angiogenesis
MMP9	Hs00957562_m1	ECM degradation, inflammation
TIMP1	Hs99999139_m1	inhibitions of MMPs, ECM homeostasis
TIMP2	Hs00234278_m1	inhibitions of MMPs, tissue remodeling
VDR	Hs01045843_m1	vitamin D signaling
NR3C1	Hs00353740_m1	glucocorticoid signaling

Supplementary Table 2. List of TaqMan probes used for quantitative RT-PCR to assess gene expression levels. For each gene, the official gene name and assay ID was provided (Applied Biosystems, ThermoFisher).
